# Myeloid Neoplasms with t(5;12) and ETV6-ACSL6 Gene Fusion, Potential Mimickers of Myeloid Neoplasm with PDGFRB Rearrangement: Case Report with Imatinib Therapy and Review of the Literature

**DOI:** 10.1155/2016/8324791

**Published:** 2016-09-26

**Authors:** Javier De Luca-Johnson, Jose I. Ruades Ninfea, Lauren Pearson, Joanna Conant, Ronald Bryant, Neil A. Zakai, Mary E. Tang

**Affiliations:** ^1^Department of Pathology and Laboratory Medicine, University of Vermont Medical Center, 89 Beaumont Avenue, Courtyard at Given S269, Burlington, VT 05405, USA; ^2^Department of Medicine, Division of Hematology and Oncology, University of Vermont Medical Center, 89 Beaumont Avenue, Given E-214-UVM363, Burlington, VT 05405, USA

## Abstract

We report the second case of ETV6-ACSL6 associated myeloproliferative neoplasm that has received a full course of imatinib therapy. The patient was a 51-year-old previously healthy man who presented with three months of worsening dyspnea and was found to have a white count of 216,000/cmm, of which 84% were eosinophil lineage. Cytogenetic analysis revealed a t(5;12)(q31~33;p13). FISH was negative for PDGFRB rearrangement but additional FISH testing demonstrated an ACSL6 rearrangement. ETV6-ACSL6 gene fusion is a rare abnormality that most often presents as a myeloproliferative-type disorder with prominent eosinophilia or basophilia. Review of the literature yielded a total of 11 previous cases. This gene fusion results in a t(5;12)(q31~33;p13) that mimics the t(5;12) found in ETV6-PDGFRB neoplasms. Identification of the fusion genes involved in t(5;12) in eosinophilia-associated myeloproliferative disorders is crucial to direct an effective treatment plan. In particular, while tyrosine kinase inhibitor therapy is effective in patients with PDGFRB rearrangement, there is little information on imatinib efficacy in patients with ETV6-ACSL6 gene fusion. Our patient was found to be nonresponsive to imatinib therapy.

## 1. Introduction

We report the second case of ETV6-ACSL6 associated myeloproliferative neoplasm that has failed a complete course of imatinib therapy and review the literature on this abnormality. ETV6-ACSL6 fusion is a rare abnormality that is associated with myeloproliferative disorders, myelodysplasia, or acute myeloid leukemia with eosinophilia or basophilia. The ETV6-ACSL6 results in a t(5;12)(q31~33;p13) that mimics the t(5;12) found in ETV6-PDGFRB neoplasms. Identification of the fusion genes involved in t(5;12) in eosinophilia-associated myeloproliferative disorders is crucial to direct an effective treatment plan. Evidence has shown sustained clinical response, molecular remission, and low toxicity profile with imatinib treatment in patients harboring PDGFRB rearrangement [1] but there is little information on imatinib efficacy in patients with ETV6-ACSL6 gene fusion.

## 2. Case Report

### 2.1. Clinical and Pathologic Findings

**Figure 1:**
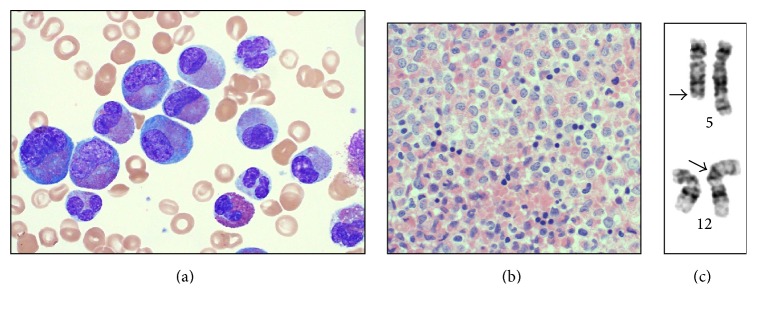
Myeloid neoplasm with t(5;12) and ETV6-ACSL6 gene fusion: morphologic and cytogenetic features. (a) Peripheral blood with a predominant population of eosinophil precursors: large cells with mononuclear, monolobated nuclei and abundant cytoplasm containing eosinophilic granules. The predominant cell type is eosinophil myelocyte, but there is a spectrum of forms including eosinophil metamyelocytes, eosinophils bands, and mature eosinophils. Promyelocytes and myeloblasts are not observed (Wright stain, 1000x). (b) Bone marrow biopsy showing replacement of the marrow space by sheets of eosinophils and eosinophil precursors (hematoxylin and eosin, 500x). (c) Partial karyogram demonstrating t(5;12)(q31;p13) that was identified in 17 out of 20 cells cultured.

A 51-year-old previously healthy man presented to the emergency room with a three-month history of shortness of breath, palpitations, intermittent cough, and unintentional 15-pound weight loss. He was treated by his primary care physician for asthma and environmental allergies but his symptoms failed to improve. Physical examination was unremarkable except for tachycardia and hepatosplenomegaly. Ultrasound of the abdomen showed hepatomegaly measuring 21 cm with normal echogenicity and an enlarged spleen measuring 18 × 13 × 8 cm. Laboratory studies revealed hemoglobin of 7.7 gm/dL, platelet count of 84 K/cmm, and WBC of 216,000/cmm with 16% eosinophils and 68% abnormal eosinophil precursors (Figure 1(a)).

He was admitted to the hematology oncology service and scheduled for emergent bone marrow evaluation. The bone marrow biopsy demonstrated a markedly hypercellular bone marrow with extensive replacement of the marrow space by sheets of eosinophils in various stages of maturation with a predominance of mid-stage eosinophil forms with abnormal vacuolation, hypogranulation, hypolobation, and nuclear-cytoplasmic asynchrony (Figure 1(b)). There was no increase in blasts, mast cells, or fibrosis (CD34, tryptase, and reticulin, resp.). The residual myeloid elements were too few in number to evaluate for dysplasia. Initial cytogenetic analysis revealed a t(5;12)(q31~33;p13) in 17 of 20 cells (Figure 1(c)) and a presumptive diagnosis of myeloid neoplasm with eosinophilia and translocation t(5;12) suggestive of PDGFRB rearrangement was made. Confirmatory testing for ETV6-PDGFRB gene fusion was negative using both a dual-fusion probe set and a PDGFRB break-apart probe (Mayo Medical Laboratories). An ETV6 break-apart probe confirmed an ETV6 rearrangement with a partner gene other than PDGFRB. FISH for FIP1L1-PDGFRA and BCR/ABL1 were also negative. In light of isolated reports of eosinophilia with t(5;12) with ETV6-ACSL6 gene fusion, additional testing with an ACSL6 break-apart FISH probe (Empire Genomics, LLC) was performed and demonstrated ACSL6 rearrangement in 80% of cells.

### 2.2. Clinical Course

**Figure 2:**
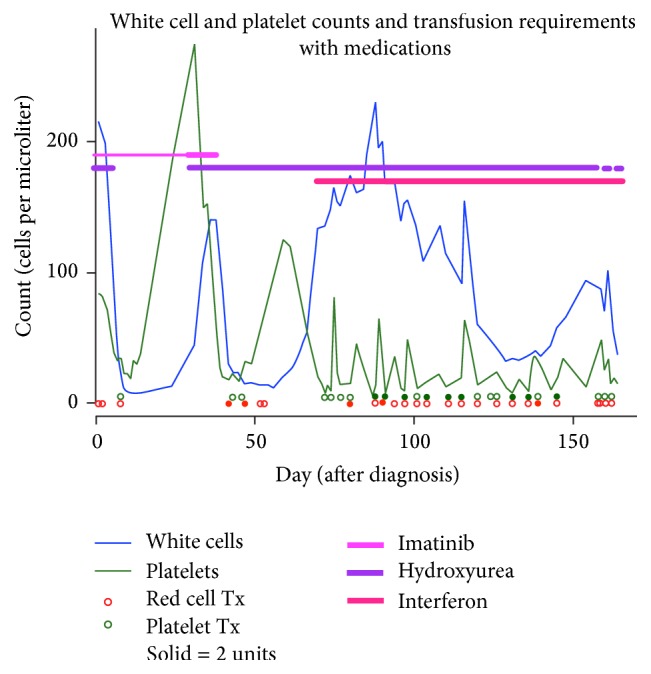
Summary of clinical course. The “Solid = 2 units” refers to the “Red cell Tx” and the “Platelet Tx.” If the red circle is solid, this indicates that 2 units of red cells were transfused. If the green circle is solid, this indicates that 2 units of platelets were transfused.

Soon after admission, with the presumptive diagnosis of myeloid neoplasm with PDGFRB rearrangement, the patient was started on imatinib 200 mg/day. Hydroxyurea 2 gm every 12 hours was added to rapidly reduce the white blood cell count. The patient's WBC, hematocrit, and platelet count decreased significantly but after 10 days of treatment he required RBC and platelet transfusions so the hydroxyurea was discontinued. After two weeks he was discharged on imatinib and initially seemed to respond to therapy. His white count reached a nadir of 8,000 and his presenting symptom of shortness of breath entirely resolved. However, by day 24 his white cell count rose to 130,000 K/cmm (Figure 2) and he was restarted on hydroxyurea. The patient received imatinib 200 mg/day for four weeks and it was increased to 400 mg/day for an additional week before the imatinib was discontinued due to lack of hematological response, characterized by progressive leukocytosis (peak WBC count 139,000/cmm), anemia (hemoglobin 7.6 gm/dL), and thrombocytopenia (platelet count 18 K/cmm). The patient was subsequently treated with hydroxyurea, prednisone, and pegylated interferon alfa and after 8 weeks the patient's WBC markedly decreased and stabilized at 30,000 K/cmm. However, the eosinophilia persisted and the patient required regular RBC and platelet transfusions. He eventually elected to discontinue transfusion support and was transitioned to hospice care, where he passed away nine months following his initial diagnosis.

## 3. Discussion

Myeloid neoplasms with t(5;12) and ETV6-ACSL6 gene fusion are rare, poorly characterized hematologic neoplasms with an aggressive clinical course characterized by eosinophilic and/or basophilic leukocytosis. Literature review reveals 11 previous cases of myeloid neoplasm with ETV6-ACSL6 gene fusion confirmed by either FISH, RT-PCR, or next-generation sequencing (NGS) with targeted RNA-seq [2–8]. Several other cases of t(5;12) with eosinophilia or basophilia may represent additional cases but confirmatory testing was not performed [5, 9, 10]. Cases with ETV6-ACSL6 gene fusion are clinically aggressive, with mean survival from time of diagnosis of less than one year. A single case demonstrated survival beyond one year [4]. Other important clinical features include male preponderance (9 of 12 cases) and eosinophilic and/or basophilic leukocytosis (12 of 12 cases). All ages are affected, with a range of 27–68 years and an average age of 47 years.

Under current guidelines ETV6-ACSL6 neoplasms are not classified as a unique disease entity. Despite a unifying molecular alteration, these patients present with a prominent eosinophilia or basophilia but may be classified as almost any myeloid neoplasm except chronic myeloid leukemia. Our case presented as chronic eosinophilic leukemia, not otherwise specified (CEL-NOS). Among the 11 previously published cases there were three cases of myelodysplastic syndrome, two cases of acute myeloid leukemia, and one of each of atypical chronic myeloid leukemia, polycythemia vera, polycythemia vera/acute myeloid leukemia overlap, myeloproliferative neoplasia, not otherwise specified, myelodysplastic/myeloproliferative neoplasia overlap, and CEL-NOS.

The etiology of the eosinophilia in ETV6-ACSL6 is not well understood. ACSL6 encodes for acyl-CoA synthetase long-chain family member 6, a protein that catalyzes formation of acyl-CoA from fatty acids. It plays a major role in fatty acid metabolism in the brain [11] and is expressed in brain, fetal liver, and bone marrow and is highly conserved [2]. It has not been associated with other neoplastic diseases [12]. ETV6 codes for a transcription factor that is commonly deleted or translocated in myeloid and lymphoid malignancies [7]. It has been implicated in leukemogenesis by generation of fusion proteins that modulate the activity of transcription factors or kinases [3]. Unlike BCR-ABL1, PDGFRA, and PDGFRB, the ETV6-ACSL6 gene fusion does not result in a fusion protein with constitutive tyrosine kinase activity. Of the four cases with molecular breakpoint analysis, all four had fusions involving the ETV6 region between exons 1 to 2 and the breakpoints on ACSL6 were highly variable, spanning from the 5′ end of the gene to the 3′ UTR. Fusion transcripts were detected in all four cases but did not result in a common transcript, making a fusion gene mechanism unlikely [2, 3].

The 5q31 region has other genes associated with eosinophilia. Telomeric to ACSL6 lies the cytokine gene cluster that includes interleukin- (IL-) 3, IL-5, and granulocyte/macrophage colony-stimulating factor (GM-CSF). These genes are the main cytokine mediators of eosinophil proliferation and differentiation [13]. Increased expression of IL-3 protein transcript has been documented in two ETV6-ACSL6 cases and dysregulation of the IL-3 gene due to a translocation event has been hypothesized as a mechanism for eosinophilia [3, 5]. The 5q31–33 region has also been linked to familial eosinophilia (EOS) [14]. Mutation screening of the IL-3, IL-5, and GM-CSF genes in EOS individuals found no mutations in these genes or in the IL-3/GM-CSF enhancer region, suggesting that EOS is not caused by a mutation in these genes but by another gene in this region [15]. ACSL6 has not been investigated as a cause of EOS.

Identification of the fusion genes involved in t(5;12) in eosinophilia-associated myeloproliferative disorders is crucial to direct an effective treatment plan. While evidence has shown sustained clinical response, molecular remission, and low toxicity profile with imatinib treatment in patients harboring PDGFRB rearrangement [1], there is no evidence for imatinib efficacy in patients with ETV6-ACSL6 gene fusion. Our patient was treated with a full course of imatinib and was found to be nonresponsive. Two other patients with ETV6-ACSL6 gene fusion have been treated with imatinib and both were found to be nonresponsive. The first patient received a short course of therapy [5], while the second patient received a full course (3 months) of therapy [8]. Specific dosage information is lacking in the prior cases.

Lack of response to imatinib is compatible with our current understanding of oncogenesis in ETV6-ACSL6 related translocation events and the lack of an obvious protein target for anti-tyrosine kinase therapy. Notably, second or third generation tyrosine kinase inhibitors have not been attempted to date and could be offered on an empiric basis with intent on targeting downstream targets. Patients presenting with eosinophilia-associated myeloproliferative neoplasm who lack overexpression of PDGFRA or PDGFRB have been imatinib-responsive [6], emphasizing that it is reasonable to begin a therapeutic trial with tyrosine kinase inhibitors in symptomatic patients pending further gene fusion characterization.

Additional therapeutic options in CEL-NOS include Mepolizumab, an investigational anti-IL5 antibody that has shown activity in patients with hypereosinophilic syndrome [16]. Although ETV6-ACSL6 neoplasms usually lack IL-5 overexpression [3], there is potential for response to Mepolizumab even when IL-5 is at physiologic levels [17]. Alemtuzumab is an anti-CD52 monoclonal antibody that has also been shown to produce hematological and clinical (no cytogenetic) responses in CEL-NOS but is reserved for patients with refractory disease because of significant adverse effects [18]. The role of bone marrow transplant has not yet been determined in CEL-NOS; case reports have described promising results and currently this appears as the only possible curative option in CEL-NOS patients [19].

In the context of eosinophilic leukocytosis, the finding of t(5;12) is highly suggestive of myeloid neoplasm with PDGFRB rearrangement. However, it is essential to confirm PDGFRB rearrangement in order to effectively direct treatment and predict prognosis. Little is known about the pathogenesis and treatment of ETV6-ACSL6 associated neoplasms. Our case is the second to demonstrate no response to a full course of imatinib therapy. Further study may elucidate the mechanism of disease and lead to effective therapy.
